# Individual Differences in Working Memory Capacity and Temporal Discrimination

**DOI:** 10.1371/journal.pone.0025422

**Published:** 2011-10-07

**Authors:** James M. Broadway, Randall W. Engle

**Affiliations:** Georgia Institute of Technology, Atlanta, Georgia, United States of America; Duke University, United States of America

## Abstract

Temporal judgment in the milliseconds-to-seconds range depends on consistent attention to time and robust working memory representation. Individual differences in working memory capacity (WMC) predict a wide range of higher-order and lower-order cognitive abilities. In the present work we examined whether WMC would predict temporal discrimination. High-WMC individuals were more sensitive than low-WMC at discriminating the longer of two temporal intervals across a range of temporal differences. WMC-related individual differences in temporal discrimination were not eliminated by including a measure of fluid intelligence as a covariate. Results are discussed in terms of attention, working memory and other psychological constructs.

## Introduction

Working memory capacity (WMC) refers to an ability to maintain, manipulate, and access mental representations as needed to support complex cognition [1], [2]. WMC varies widely across individuals and reliably predicts higher-order cognitive abilities such as novel problem solving or general fluid intelligence (*gF*) [1], [3]–[5]. WMC also predicts lower-order abilities reflected in the accuracy and/or latency of simple decisions, especially under strongly interfering conditions such as: looking away from a peripheral sudden-onset stimulus (antisaccade task) [6], or naming the ink-color of an “incongruent” color-word (Stroop task) [7]. Such tasks as these require cognitive control to over-ride the more automatic but incorrect response. In contrast, individual differences in WMC are not often associated with performance in tasks where the more automatic response is instead the correct one: such as looking toward a sudden-onset stimulus in a prosaccade task or naming the ink-color of a “congruent” color-word in a Stroop task [1], [6], [7].

The “executive attention view” of WMC [1], [8] emphasizes the supervisory role(s) of the “central executive” construct in Baddeley's influential multiple-component model of working memory [9] more than the “phonological loop,” “visuospatial sketchpad,”or various buffers that have been much the focus of much other research into individual differences in WMC (see [10]). According to this perspective, WMC is not directly about remembering per se, but instead reflects a more general ability to control attention and exert top-down control over cognition. The domain-general ability to combat interference through the control of attention is also proposed to account for strong relationships between WMC and other important abilities like *gF* [1], [3], [8]. There is a consensus that WMC depends on a ubiquitous frontal-parietal brain network implicated across a range of experimental tasks requiring cognitive control [11]–[13].

However, strongly interfering conditions are not always *sufficient* to observe WMC-related individual differences, as when searching for a “conjunction target” among highly similar distractors [14]. And strongly interfering conditions are not always *necessary* to observe WMC-related individual differences, as when counting a small number of objects [15], [16], or maintaining psychomotor vigilance [17]. Recently we have reported that individual differences in WMC predict yet another core mental ability important for the control of behavior, time estimation [18], assessed by the method of temporal reproduction.

This outcome was consistent with recent theories of short-term memory [19]–[21], and of individual differences in WMC [22], that have likened recall and recognition to acts of perceptual discrimination, but made on the basis of multiple dimensions represented in memory- among the most salient of which is temporal. Theoretical connections between time perception and WMC are considered next in more detail.

### Timing and WMC

#### Dual-component WMC theory

Recently Unsworth and Engle [22] introduced a “dual-component framework” for understanding individual differences in WMC that has much in common with general theories of short-term memory; in particular with those proposing that recall and recognition depend much on discriminating temporal relations among events [19]–[21]. According to the WMC theory of Unsworth and Engle [22], WMC reflects the interaction of two components, “primary memory” (likened to the “focus of attention” in other theories [23], [24] and “secondary memory” (associative memory [25] outside the focus of attention), functioning together to support active maintenance and selective retrieval. Selective retrieval depends in large part on the specificity of “search sets” in secondary memory, which are delimited in large part by temporal-contextual cues [22]. Unsworth and Engle proposed that individuals differ in performance across a wide range of memory tasks (e.g., serial order recall as well as free recall) in large part because low-WMC individuals are less able than high-WMC to use “temporal-contextual cues” that support efficient search of secondary memory [22].

According to this account, a complete session of memory testing (e.g., for letter strings) forms a hierarchy of nested temporal contexts. Temporal-contextual elements associated with each level undergo change at different rates. For example the experimental session is the highest, the global-level context. Contextual elements associated with the global-level context change relatively slowly. Below this level in the hierarchy, a complete list of items to be recalled in a single trial is intermediate, the list-level context. Contextual elements associated with the list-context change more rapidly: within a single global-context, the participant is exposed to a number of different lists. Below this level, individual items in a particular list constitute the lowest, the item-context. Contextual elements associated with the item-context change most rapidly: within a single list-context, the participant is exposed to a number of different items. The dual-component WMC theory proposes that low-WMC individuals are less able to use information that distinguishes these temporal contexts with sufficient precision to prevent confusion of search-sets, which leads to greater forgetting and erroneous recall.

Confusion of information *within* a contextual level is common, as seen for example in *transposition gradients* for items swapped during recall output for a particular list. Notably, adjacent items are most likely to be swapped [19]–[22]. Confusion of information *across* contextual levels is also common, and shows a systematic property giving additional insight into similarity-based mechanisms of forgetting. For example, *previous-list intrusions* occur in serial and free recall tasks when the participant incorrectly recalls an item that appeared in the list previous to the one currently tested. Importantly, it is most often the case that the incorrectly reported item had appeared in the same within-list position as the item from the current list for which it is swapped [19]–[22].

Like the OSCAR model of short-term memory [21], the dual-component WMC theory explains previous list intrusions by the confusability, or similarity, of the temporal contexts in which the two swapped items had appeared during list learning [22]. Notably, low-WMC individuals are more likely to commit previous list intrusions in serial order and free recall tasks [26]; suggesting that for low-WMC, memory search sets are not well-constrained to include only representations of items from the current list being tested. This provides evidence, albeit somewhat indirectly, for greater confusion of temporal contexts by low-WMC individuals. This is also consistent with predictions that could be generated from the OSCAR model [21].

Notably, recent fMRI experiments have shown common activation in prefrontal cortex when retrieving temporal context information across diverse stimulus domains [27]. This is overall consistent with assumptions that WMC depends on (a) prefrontal cortex [13] and (b) retrieving temporal context information [22]. The dual-component framework proposed by Unsworth and Engle would seem to directly predict that individual differences in WMC are associated with individual differences in temporal discrimination. The main goal of the present work was to address this question.

#### Timing theories

From the literature on time perception there are a number of theoretical links to attention and memory, as well as proposed neural substrates for these cognitive systems. There are a staggering number of time perception theories [28], but the modal frameworks broadly conform to “clock-counter” models (or pacemaker-accumulator models, e.g., [29]–[33]; see e.g., [34]–[37] for discussion of major alternatives). Most prominent among these is the *scalar expectancy theory* [29], an information-processing model originally developed to account for animal conditioning by temporal regularities in the environment.

Clock-counter models assume that event timing is accomplished through the cooperation of internal clock, memory, and decision-making components. The clock emits pulses that are transmitted to a counter (or accumulator). In the attentional-gate theory [31], [32] a gate between the clock and counter is opened and pulses are allowed to accumulate when attention is directed to judging time. More elapsed time is represented by more pulses in the accumulator. The current pulse count is continuously integrated and transferred to working memory as a single value, to be compared to the value of a sampled duration represented in “reference memory” (long-term memory). A temporal decision is made when comparison between the current pulse-count to the remembered one exceeds a threshold ratio.

Scalar expectancy theory accounts for a wide range of behavioral findings [30], including the Weber's Law property of time estimation; in which temporal judgment error increases proportionally with the length of the interval to be timed (this is often called the “scalar property” in the timing literature). However, recently there has been a major push to incorporate somewhat greater “biological plausibility” into timing models [30], [34], [37]. In a connectionist implementation of scalar expectancy theory proposed by Church and Broadbent [38], [39] durations are coded by the phase relations among signals generated by banks of multiple oscillators. Notably, the same multiple-oscillator mechanisms are proposed by the OSCAR model to underlay short-term memory [21].

Lustig, Matell, and Meck [40] traced several striking correspondences between recent computational models for timing [37] and working memory [41]; each theoretically driven by dopamine and by the activity of circuits connecting fronto-parietal cortex with subcortical basal ganglia and striatum. In broad outlines, Lustig and colleagues suggested that the identities and temporal properties of to-be-remembered events could be coded simultaneously by the same brain networks. Identity information is determined by *which* cortical neuron population fires in an oscillatory manner to encode and maintain working memory for an event. Temporal information is determined by the phase relations between such oscillatory activity, as determined by a “coincidence detection” mechanism of the striatum [37], [40] (see also [35]).

All together, theories of working memory and time perception reviewed above provide strong reasons to expect an association between individual differences in WMC and temporal judgment. Next we consider some of the existing evidence for such an association.

### Individual Differences in WMC and Timing

There have been many experiments examining effects on time estimation from imposing additional non-temporal loads on working memory and/or attention in dual-task procedures, generally finding that directing attention away from time leads to shortened time estimates and/or more variable time estimates [42]. Loads placed on verbal or visual working memory resources have shown analogous effects, independently of stimulus modality [43]. Such manipulation of available attentional or working memory resources can be “mimicked” by naturally occurring variation among individuals [1]. Systematic inter-individual variation may then become an object of measurement for psychology, in order to better understand the operation of the underlying system(s).

There have been relatively few studies that have examined relationships between individual differences in WMC and timing within the population of healthy younger adults [18], [44]–[46]. This question has been addressed to a greater extent in developmental and neuropsychological research. The picture is not always clear, but there seems to be evidence for changes temporal judgment throughout the lifespan, comparing children or older adults to younger adults [47]–[52]. Notably, tend to differ in WMC as well [51], [53], and some research appears to show associations among WMC, timing, and aging [51], [52].

Additionally, temporal processing deficits have been shown in a wide variety of neurological disorders [54]; most notably among patients with dopaminergic disorders like schizophrenia [55], [56] and Parkinson's disease [57]–[60]. These groups are known for WMC deficits as well, and some research has shown associations among timing, WMC, and schizophrenia [55] or Parkinson's disease [57], [58]; while other research has not [56].

The variegated picture that emerges for relationships among timing, WMC, and individual differences in developmental or neurological state, is likely due to the diversity of tasks and time scales used to measure temporal judgment [28], [33]. Another reason may be our general lack of knowledge about the relationship between individual differences in WMC and timing within the typical control group, i.e., healthy younger adults.

#### General fluid intelligence

Rammsayer and colleagues have extensively examined temporal processing as a predictor of *gF* [46], [61]–[62]. As noted earlier, WMC is also widely recognized as a major predictor of *gF* [1], [5]. According to the *temporal resolution power* hypothesis of Rammsayer and colleagues, faster rates of neural oscillation lead to faster information processing (see also [5], [63]); and also to better coordination of information processing. Higher temporal resolution is thus proposed to lead to better performance on WMC and *gF* tests because critical information is less likely to be lost or degraded during elementary processes supporting e.g., serial order recall or abstract problem solving [46]. Troche and Rammsayer [46] showed through structural equation modeling that WMC, temporal discrimination, and *gF* are strongly inter-related. Indeed, WMC fully mediated relationships between time perception and *gF* [46]. We sought to further examine relationships among WMC, timing, and *gF* using a temporal discrimination task designed from a signal detection theory approach [64].

### Present Research

With relatively little prior evidence in this area, an extreme-groups design can be justified for the goals of the present research [65]. Participants were identified as either high-WMC or low-WMC in a pre-screening session in which two valid measures of WMC were administered. The present method of forming extreme-groups (described in more detail below) is not unlike methods commonly used when studying cognitive effects of aging or neuropathology. We additionally obtained *gF* measures for participants (from their participation in other studies in the lab), in order to assess contributions from *gF* to relations between WMC and temporal judgment.

We predicted generally that high-WMC observers would be more sensitive to differences between temporal durations. Following the report by Troche and Rammsayer [46], in which WMC completely mediated relationships between temporal discrimination and *gF* in structural equation modeling, we predicted that WMC and *gF* would both account for variance in temporal discrimination– but if WMC-related differences in temporal discrimination were to be attenuated by including *gF* as a covariate, they would still not be completely removed.

## Methods

### Ethics Statement

The present research was conducted with approval by the Institutional Review Board of the Georgia Institute of Technology. Participants gave written informed consent.

### Participants

A total of 52 individuals (27 high-WMC, 15 women; 25 low-WMC, 16 women) participated in the present experiment. Participants were the same as in a recent study of WMC and temporal reproduction [18]; Experiment 2]. Temporal discrimination results in the present article were not reported therein. Participants were recruited from the Atlanta community or undergraduate research pool, were between the ages of 18 and 35 years (*M = *23.6, *SD = *3.9), and were compensated with a check or partial course credit.

We had measures of *gF* for 44 participants (high-WMC, *n* = 22; low-WMC, *n* = 22) from their participation in other studies in the lab. The following results include data from only these 44 participants so that *gF* could be included as a covariate in ANCOVA (ANOVA results from the full sample were similar to those from the reduced sample). WMC groups in this sample were not statistically different in age, *t* (42) = 1.65, *p = *.106 (Low-WMC *M = *24.36 years, *SD = *3.81; High-WMC *M = *22.59 years, *SD = *3.23). WMC measurement and participant recruitment procedures are described below.

### Procedure

Participants in the experiment were recruited after first visiting the lab for WMC measurement in a session lasting approximately 60 minutes. Participants performed computer-administered tasks seated at a comfortable distance from the monitor, alone in a sound-attenuated room. Participants were made aware they would be monitored for compliance with general instructions via closed-circuit cameras when the research assistant was absent from the room. All tasks in the present studies were programmed in e-prime experimental software, with presentation timing accurate to 1 millisecond [66]. The WMC tasks administered in the pre-screening session have been extensively validated as measures of domain-general WMC and executive control of cognition [67].

*Operation Span* is a test of WMC for verbal material. Participants solved simple math equations, in between encoding to-be-remembered letters presented sequentially in the center of the screen (from the set: F, H, J, K, L, N, P, Q, R, S, T, Y). Participants were prompted to report the presented letters in order after 3–7 of these equation-letter events (set-size; randomly determined on each trial), by clicking with the mouse on their choices from a 4×3 grid presenting the complete set of 12 letters that could be shown. In order to maintain correct serial position in the response sequence for recalled letters, participants were instructed to click a “blank” option for any letters they could not recall [68].

*Symmetry Span* is a test of WMC for visual-spatial material. Participants judged whether black-and-white images were symmetrical, in between encoding the location in which a red square sequentially appeared in a 4×4 grid. Participants were prompted to report the square locations in order after 2-5 of these symmetry- square events, by clicking on their choices in the cells of a 4×4 grid. In order to maintain correct serial position in the response sequence for recalled square locations, participants were instructed to click a “blank” option for any square locations they could not recall [69].

There were three trials for each set-size in each WMC task. Scoring was done automatically by the computer program. One point was assigned for each item correctly reported in correct serial position. “Strict” serial position scoring was applied, i.e., if the letters *JRKT* were to be reported, the response “JRK” would be assigned 3 points, the response “*blank* RKT” would be assigned 3 points, but the response “RKT” would be assigned 0 points. This scoring method has been shown to yield WMC scores with good reliability and validity (Conway et al., 2005). The ranges of possible scores were (0, 75) for Operation Span and (0, 42) for Symmetry Span.

Scores for each WMC task were converted to z-scores in reference to distributions of scores obtained over a period of several years of testing student and community volunteers (ages 18 to 35 years). At the time the present studies were conducted there were approximately 2,000 scores in the reference distributions for the two WMC tasks. The z- scores for the two WMC tasks were averaged to form a composite WMC z-score. Individuals were classified as high-WMC (or low-WMC) if their composite z-score fell within the upper (or lower) quartile of a reference distribution of composite z-scores. Raw score reference distribution summary statistics at the time the study was conducted were: Operation Span: *M = *57.87, *SD = *13.27, Symmetry Span: *M = *27.89, *SD = *8.67. The correlation between WMC measures was statistically reliable in the normative sample, *r = *.56, *p<*.001.

It is necessarily the case that differences in measured WMC between high- and low-WMC groups in the present experiments were statistically significant because participants were classified as high-WMC or low-WMC based on extreme composite z-scores, located in either the upper or lower tails (respectively) of a reference distribution of composite z-scores. For the sake of completeness, we report that measured-WMC was statistically different between high- and low-WMC groups, *t* (42)* = *−11.63, *p<*.001 (high-WMC *M = *.895, *SD = *.394; low-WMC *M = *−1.064, *SD = *.707; WMC scores are reported in z-score units). Recruited participants returned to the lab to perform the temporal discrimination task in the first half of a follow-up session lasting approximately 60 minutes.

#### Temporal discrimination task

The temporal discrimination task conformed to a so-called “roving” 2-alternative forced-choice task (2-AFC) [64]; see also [33] for a discussion of this design applied to temporal discrimination. In roving 2-AFC designs the “standard” interval is not necessarily a fixed duration nor is it always presented first—here, the “standard” and comparison intervals can both vary trial-to-trial [33]. Also in roving 2-AFC designs the difference between comparison intervals can be held constant while their absolute magnitudes can vary over a relatively wide range [64].

Absolute durations of paired comparison intervals and their corresponding duration differences in the present discrimination task are presented in Table 1. The difference between comparison intervals (duration difference) in the present task was 250 ms, 500 ms, or 750 ms on each trial, randomly determined. Absolute durations of comparison intervals were multiples of the shortest comparison intervals (250 ms); the longest absolute duration was 2750 ms. These time scales were chosen to assess temporal judgment over a range thought to be within the so-called “psychological present” [50], [70]. Furthermore this range covers much of the time scale at which working memory processes are thought to be critical to ongoing cognition and action [2].

**Table 1:** Paired comparison intervals and duration differences in the temporal discrimination task.

Duration Difference (ms)	Comparison Intervals (ms)	
250	250	500
	500	750
	750	1000
	1000	1250
	1250	1500
	1500	1750
	1750	2000
	2000	2250
	2250	2500
	2500	2750
500	250	750
	500	1000
	750	1250
	1000	1500
	1250	1750
	1500	2000
	1750	2250
	2000	2500
	2250	2750
750	250	1000
	500	1250
	750	1500
	1000	1750
	1250	2000
	1500	2250
	1750	2500
	2000	2750

Participants were exposed to two comparison intervals in sequence and were prompted on each trial to press the ‘b’ key if the comparison interval presented first was the longer one or the ‘n’ key if the comparison interval presented second was the longer one. The word “interval” in capital letters appeared on the screen during each comparison interval. The longer comparison interval was presented first on half of the trials, randomly determined. The stimulus defining the first comparison interval was preceded by a fixation cross for 250 ms. After the first comparison interval terminated, participants were prompted to press ‘Enter’ to view the stimulus defining the second comparison interval, which appeared after an unfilled blank-screen delay of 500 ms and a second fixation cross for 250 ms. Thus, a minimum delay of 750 ms separated the first and second comparison intervals. After the second comparison interval terminated, participants pressed ‘Enter’ to immediately view the next screen prompting their response to indicate which of the two comparison intervals had been the longer one. No feedback was provided. Temporal discrimination responses were followed by an inter-trial interval of 1000 ms. Giving participants self-paced control over the onsets of trials (and comparison intervals within trials) was intended to ensure that participants were paying full attention to the task at the onsets of stimuli defining the comparison intervals. There were 80 trials for duration difference = 250 ms, 72 trials for duration difference = 500 ms, and 64 trials for duration difference = 750 ms. Trial types were randomly intermixed so that any duration difference and any pair of comparison intervals listed in Table 1 could be experienced on a given trial.

#### Ravens Matrices

From their participation in other studies in the lab, we obtained in a post-hoc manner scores from two closely related measures of *gF* for different sub-sets of the participants in the present study. For one sub-set of participants (high-WMC *n = *11; low-WMC *n = *12), we had scores from a 12-item set of Raven's Matrices problems [71]. Participants in this task had 5 minutes to complete 12 items. Participants selected by mouse click, from an array of choices shown at the bottom of the computer screen, the figure that would best complete an incomplete abstract pattern. For a different sub-set of participants (high-WMC *n = *11; low-WMC *n = *10), we had scores from an 18-item set of Raven's Matrices problems. Participants in this task had 10 minutes to complete 18 problems. Stimulus presentation, response collection, and scoring procedures were the same as in the 12-item test. One point was assigned for each correct response, making the possible range of scores (0, 18).

To facilitate combining data across test versions, raw scores from the 12-item and 18-item Raven's Matrices tests were converted to proportion-correct scores. The two Raven's measures have been shown to strongly correlate to the same measures of WMC used in the present work, in previous large-sample studies [69], [72]; and with overlapping ranges of magnitude (For the 12-item test [69]: Operation Span *r = *.49, Symmetry Span *r = *.51. With two separate samples [72], for the 18-item test: Operation Span *r*s* = *.42 and .50, Symmetry Span *r*s* = *.56 and .62).

## Results

### Sensitivity

Correct responses increased, and errors decreased, monotonically for both WMC groups, as duration differences increased (Table 2). Correct responses were treated as “hits” and errors as “false alarms” (FA) in order to express discrimination sensitivity as *d*', which is a dependent measure from signal detection theory [64]. This measure expresses in standard deviation units, how distant was a person's sensitivity to differences between stimuli, from the point of perfect indifference (represented by zero); after controlling for “guesses” or “response bias” (a predisposition to say e.g., “second one longer”). Higher *d*' means that there was greater sensitivity to differences between stimuli. Furthermore, *d*' allows sensitivity across a range of stimulus differences to be expressed in a common metric [64]. Proportion-correct (p (Hit)) and proportion-error (p (FA)) scores for each individual were converted to probabilities according to Table A5.1 in [64], changing “p (Hit)” and “p (FA)” into “*z-*Hit” and “*z-*FA” respectively [64]. Then *d*' was calculated according to equation 7.2 for 2-AFC designs in [64]: *d*' = (1√ 2) * (*z-*Hit–*z-*FA).

**Table 2:** Means (Standard Deviations) for proportions of correct responses (hits) and errors (false alarms) by high-WMC and low-WMC in temporal discrimination across duration differences.

	250 ms			
	p (Hit)		p (FA)	
High	.806	(.076)	.195	(.079)
Low	.687	(.085)	.313	(.085)
	500 ms			
	p (Hit)		p (FA)	
High	.919	(.074)	.081	(.074)
Low	.805	(.098)	.197	(.098)
	750 ms			
	p (Hit)		p (FA)	
High	.949	(.049)	.051	(.049)
Low	.850	(.109)	.150	(.109)

Note. N = 44.

**Figure 1:**
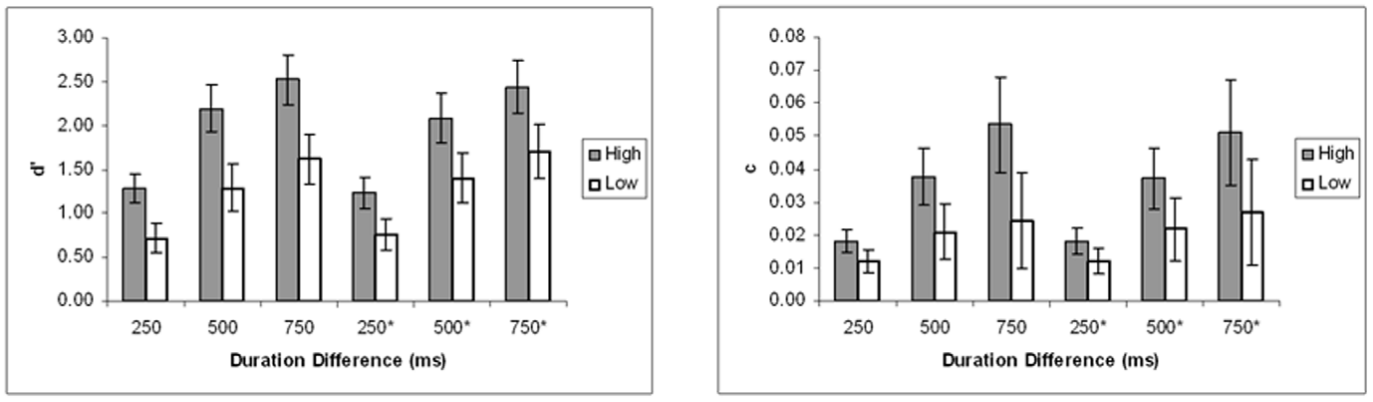
Temporal discrimination by WMC groups. Left panel: Temporal discrimination sensitivity (*d*') at three duration differences (not starred) and with *gF* as covariate (starred). Right panel: Response bias (*c*) at three duration differences (not starred) and with *gF* as covariate (starred). Error bars represent 95% confidence intervals. Legends refer to WMC groups.

Discrimination sensitivity increased monotonically for both WMC groups as duration differences increased. High-WMC observers were better able to discriminate the longer of two temporal intervals than low-WMC across the range of duration differences. See Figure 1 (A; not starred). A 3 (Duration Difference: 250 ms, 500 ms, 750 ms) ×2 (WMC: High, Low) mixed-model ANOVA was applied to mean *d*'. (All ANOVA statistics were Huynh-Feldt corrected as necessary for any violations of sphericity). The main effect of duration difference was statistically significant, *F* (2, 41) = 147.79, *p*<.001, η*_p_* ^2^ = .779. The main effect of WMC was significant, *F* (1, 42) = 26.26, *p*<.001, η*_p_* ^2^ = .385. These effects were qualified by the significant interaction of duration difference with WMC, *F* (2, 41) = 4.94, *p* = .009, η*_p_* ^2^ = .105.

Inspecting the means for *d*' suggests the interaction was due to the smaller difference between WMC groups for the 250 ms duration difference (*Mean Difference = *.56, *SD = *.12) compared to the larger differences between WMC groups for the 500 ms and 750 ms duration differences (respectively *Mean Difference = *.92, *SD = *.19; and *Mean Difference = *.92, *SD = *.19); although high-WMC individuals were more accurate overall.

#### Correlations

Spearman's rank-order correlations between *d*' and response time (RT) across levels of duration difference were consistently negative (Table 3), suggesting there were no speed-accuracy tradeoffs. Spearman's rank-order correlation coefficients were examined due to the extreme-groups nature of the sample. WMC was binary coded (low-WMC = 0, high-WMC = 1). Faster responses were generally associated with more accurate temporal discrimination (and also with higher WMC). Additionally, given the relatively wide range of time scales tested, we sought also to evaluate whether measured levels of performance could be considered to reflect the same construct of temporal sensitivity across conditions. Spearman's rank-order correlations for *d*' among the three duration difference conditions were consistently high and statistically significant (Table 3). These results show that the rank ordering of individuals by discrimination sensitivity was very consistent across different levels of absolute duration and duration difference, suggesting that the same construct of temporal sensitivity was measured across the ranges of duration differences and comparison intervals. Furthermore, Cronbach's alpha reliability was computed for the task, defining “test item” by either the first or second comparison interval of each pair (e.g., 1250 ms). When test item was defined by the first comparison interval, *r = *.923, *p<*.001; and when defined by the second comparison interval, *r = *.926, *p<*.001. These results show a high degree of “internal consistency,” i.e., systematic variance shared among all responses to all comparison interval pairings in the task, irrespective of absolute duration or order of comparison intervals.

**Table 3:** Rank-order correlations among gF, WMC, and temporal discrimination variables.

		1.	2.	3.	4.	5.	6.	7.	8.	9.	10.	11.
1.	Raven	-										
2.	WMC	.541**	-									
3.	*d*' 250	.413**	.610**	-								
4.	*d*' 500	.490**	.592**	.904**	-							
5.	*d*' 750	.418**	.597**	.873**	.864**	-						
6.	*c* 250	.225	.486**	.793**	.761**	.724**	-					
7.	*c* 500	.250	.364*	.549**	.619**	.531**	.398**	-				
8.	*c* 750	.303*	.375*	.348*	.431**	.418**	.399*	.533**	-			
9.	RT 250	.025	−.279	−.302*	−.240	−.213	−.356**	−.119	−.127	-		
10.	RT 500	−.111	−.369*	−.353*	−.347*	−.289	−.522**	−.182	−.253	.789**	-	
11.	RT 750	.037	−.365*	−.423**	−.389**	−.347*	−.540**	−.303*	−.348*	.706**	.866**	-

*Note.* N = 44. Spearman's rank-order correlation coefficients were examined due to the extreme-groups nature of the sample. WMC was binary coded here (low-WMC = 0, high-WMC = 1). *Raven* represents proportion-correct scores for Raven's Matrices tests.

**p<.01.

*p<.05.

### Bias

One of the proposed virtues of *d*' as a measure of discrimination sensitivity is that it controls for any bias in favor of one response versus the other (a predisposition to say e.g., “second one longer”) [64]. Still, it is customary to estimate response bias for the sake of completeness. Bias was calculated here as *c* (for *criterion*; [64]), because unlike other measures of response bias such as β, estimates of *c* are not affected by estimates of *d*', and vice-versa [20]. *C* was calculated according to equation 2.1 in [64]: *c = *−1/2 * (*z-*Hit + *z-*FA). *C* expresses in standard deviation units how far a participant's response criterion was located from the point of perfect indifference (represented by zero). Macmillan and Creelman [64] note that in simple detection tasks in which a participant must simply report whether a target is present or absent, higher *c* generally indicates a more conservative setting (less willingness to say “yes, target present”). However, they observe that *c* has no reasonable interpretation in a 2-AFC design like the present task. Therefore, we will confine interpretation to the measure of sensitivity, *d*'.

While zero response bias might be “ideal,” it is not reasonable to expect it in practice. The following results show that the response criterion was indeed not located at zero for most participants for discriminating the 250 ms duration difference, and moved farther from zero with increasing duration difference; and was farther from zero overall for high-WMC individuals compared to low-WMC. A 3 (Duration Difference: 250 ms, 500 ms, 750 ms) ×2 (WMC: High, Low) mixed-model ANOVA on mean *c* showed that the main effect of duration difference was significant, *F* (2, 41) = 15.50, *p*<.001, η*_p_* ^2^ = .431, as was the main effect of WMC, *F* (1, 42) = 8.37, *p*<.001, η*_p_* ^2^ = .261. The interaction of duration difference and WMC did not reach statistical significance, *F* (2, 41) = 3.12, *p* = .058, η*_p_* ^2^ = .069. See Figure 1 (B; not starred).

### gF as Covariate

#### Sensitivity

Unsurprisingly, the difference in *gF* between WMC groups was statistically significant, *t* (42)* = *−3.897, *p = *.001 (high-WMC *M = *.718, *SD = *.177; low-WMC *M = *.489, *SD = *.210), and *gF* was correlated with *d*' (Table 3), justifying the following ANCOVAs (*gF* was evaluated in the following models as *Raven*'*s proportion-correct* = .604). (Correlations between *gF* and temporal discrimination variables were of somewhat larger magnitude for the 18-item version of Raven's Matrices than for the 12-item version, unsurprisingly. However, because the correlations involving data from the two versions were statistically significant and in the same direction, we do not believe this harms the overall validity of the analyses.)

As in the ANOVA, the main effect of duration difference was statistically significant, *F* (2, 40) = 8.37, *p*<.001, η*_p_* ^2^ = .170. See Figure 1 (A; starred). As in the ANOVA, the main effect of WMC was significant, *F* (1, 41) = 12.72, *p* = .001, η*_p_* ^2^ = .237. Unlike in the ANOVA, the interaction of duration difference with WMC was not significant, *F* (2, 40) = 1.85, *p* = .164, η*_p_* ^2^ = .043. The main effect of *gF* did not reach statistical significance, *F* (1, 41) = 3.38, *p* = .073, η*_p_* ^2^ = .385; neither did the interaction of duration difference with *gF*, *F* (2, 40) = 1.55, *p* = .220, η*_p_* ^2^ = .036. However, *gF* was positively and significantly correlated with *d*' for all three duration differences (Table 3).

#### Bias

Unlike in the ANOVA, the main effect of duration difference was not significant, *F<*1. See Figure 1 (B; starred). As in the ANOVA, the interaction of duration difference and WMC was not significant, *F* (2, 40) = 1.40, *p* = .243, η*_p_* ^2^ = .033. As in the ANOVA, the main effect of WMC was significant, *F* (1, 41) = 8.41, *p* = .006, η*_p_* ^2^ = .170. The main effect of *gF* was not significant, *F<*1; neither was the interaction of duration difference with *gF, F<*1. However, *gF* was positively correlated with *c*, and significantly so for the largest duration difference (Table 3).

## Discussion

The present work adds to a small number of studies so far to examine relationships among individual differences in WMC, *gF*, and temporal judgment, within the population of healthy younger adults. Low-WMC individuals were less sensitive than high-WMC at identifying the longer of two comparison intervals across a range of absolute durations and duration differences. WMC-related effects on temporal discrimination were not eliminated by including *gF* as a covariate. There was an interaction between duration difference and WMC in ANOVA, but this was removed by including *gF* as covariate in ANCOVA. Therefore we conclude the interaction between WMC and duration difference was due to variance shared between WMC and *gF* and only apparently related to WMC. Overall, results support the idea of close relations between WMC and time perception over and above any shared relations with *gF*, consistent with a limited amount of previous related work [11], [18]. Overall, results are consistent with predictions from a recent theory proposing that individual differences in WMC are closely related to the ability to discriminate events by their temporal relations [32], [33]; and with predictions from general theories of short-term memory that attribute recall and recognition to mechanisms of temporal discrimination [30] and/or temporal context-reinstatement [31].

The present work raises questions concerning the degree to which the relationship between WMC and timing depends on executive control of attention versus the ability to robustly encode, maintain, and access representations in working memory. This is a difficult problem to tease apart, given the shared dependence of attention and working memory on a ubiquitous, dopamine-driven fronto-parietal network [11]-[13], [30], [37], [39], [59], [60]. Also Engle and colleagues have argued elsewhere that these functions might not be strictly separable [1, 8. 22]. For example Unsworth and Engle [22] proposed that WMC emerges from ongoing interactions between a flexible focus of attention [23], [24] or “primary memory” that provides direct access to a limited number of representations for immediately past events, and an associative memory or “secondary memory” that is the substrate of controlled retrieval of representations that have been displaced from primary memory [25].

In the present work the timing task required a comparison between a currently elapsing time interval and a memory representation of an interval that just finished elapsing. We propose that low-WMC individuals would be more likely to experience lapses of attention to currently elapsing time; and where multiple durations are tested as in the present task, would experience greater confusion among representations for time already elapsed. Teasing apart these contributions to overall performance is an important direction for future research.

Matters are complicated somewhat by the unresolved general question of which way the causal arrow should point between the constructs of time perception and memory. Some timing theories invoke a comparison to remembered duration to explain interval timing, e.g., [29]. Other timing theories propose that the experience of time arises due to time-varying decay of memories [35], [36]. And as noted earlier, some memory theories explain recall and forgetting by discrimination of temporal and other attributes [19], [20] or re-instatement of temporal-contextual cues [21].

Given that memory and time are intrinsically confounded (i.e., all events that are remembered or forgotten are by definition events that occurred in the past), it has remained a difficult problem for the researchers to determine empirically whether memory “causes” the experience of time or vice-versa. Most often, the direction of the causal arrow is decided by assumption. The present study shows that individual differences in WMC and temporal discrimination are associated but much additional work is needed, perhaps using structural equation modeling or finer-tuned experimentation, to disambiguate the causal direction of this relationship.

The present work adds to the few studies that have shown that WMC, *gF*, and timing are strongly associated abilities in the population of healthy younger adults [18], [46]. But the separable contributions of WMC and *gF* to timing remain unclear. Indeed the question might be more tractable if posed otherwise: treating timing instead as one of the “primitives,” along with WMC, for explaining *gF* (see, e.g., [5]). The goal of including a measure of *gF* as a covariate in the present work was mainly to examine whether the relationship between WMC and timing could be explained by a third variable that is correlated with each of them. Because WMC and timing each share much of their systematic variance with *gF* [46], *gF* appeared to be the strongest candidate for this purpose. Correlations indeed showed strong relationships among the three variables, but still ANCOVA demonstrated a strong relationship between WMC and timing, over and above any variance shared with *gF*. However, the ANCOVA did remove the interaction between WMC and duration difference that had been significant in the ANOVA. For reasons that are discussed below, this interaction is difficult to interpret but apparently it was associated with variance in *gF* not WMC. That said, we might speculate within clock-counter terms that WMC contributes to temporal judgment via the control of attention directed to elapsing time, and the controlled retrieval of memory representations for elapsing time; while *gF* contributes to timing through the decision process that compares the currently elapsing interval to the elapsed interval represented in memory. A prominent alternative to this suggestion is discussed next.

Troche and Rammsayer [46] proposed that *temporal resolution power* is fundamental to both WMC and *gF*. According to this view, faster rates of neural oscillation (reflected in finer temporal discrimination) lead to better performance on WMC and *gF* tests because critical information is less likely to be lost or degraded during elementary cognitive processes supporting e.g., serial order recall or abstract problem solving (see also, e.g., [5], [63]). Their best-fitting structural equation model showed that WMC completely mediated the relationship between timing and *gF*– meaning that whatever variance in *gF* that was explained by temporal judgment, that same variance and more was explained by WMC. The authors interpreted this result as consistent with the theory that timing is the causal primitive for WMC, which then “causes” *gF*. However, the result is also consistent with the alternative view that WMC is the more fundamental variable for both temporal processing and *gF*. The authors did not test an alternative structural equation model to evaluate the fit of this alternative perspective. This would be clear direction for future research.

It should also be noted that the present work does not show that WMC-related differences in discrimination are specific to temporal processing per se. Future work in this area is much needed that would include also tests of discriminating non-temporal stimulus attributes such as brightness or line-length. This would help evaluate whether WMC-related differences observed here do not arise from a general ability to form and maintain representations, and/or to discriminate them. Indeed it appears that temporal and non-temporal discrimination are also strongly associated with each other and with *gF* [73]. Parceling out the shared and unique relationships to WMC among these constructs should be a focus of future research.

An important limitation of the present design is that the duration differences were not proportionally scaled to the absolute durations of the comparison intervals, to respect Weber's Law for timing. Thus in the set of durations used here, the smaller duration differences were more often a smaller proportion of the comparison intervals. This confound could have contributed to increases in discrimination sensitivity across increasing duration differences; however, this would not appear to compromise the overall difference in sensitivity between WMC groups. Likewise the interaction of duration difference with WMC is difficult to interpret on account of this problem, but again this would not appear to compromise the overall difference in sensitivity between WMC groups– especially since this interaction was removed in the ANCOVA and therefore seems to have been related more specifically to *gF*.

A related consequence of the design is that many of the durations were probably long enough to support a strategy of chronometric counting. This is of concern here because individual differences in WMC are also associated with differences in enumeration [15], [16]. However, given the wide range of durations and unpredictability of their selection on each trial, we feel it is unlikely that participants deployed an effective counting strategy with any consistency. Still, relationships between individual differences in WMC, counting, and temporal processing would seem to be a ripe area for future research, especially given relationships between counting and timing [74], [75], WMC and counting [15], [16], WMC and timing [18], [46]; and the general importance of *ordinal* as well as temporal coding for working memory [76], [77].

### Summary and Conclusions

In the present experiment, individual differences in WMC predicted differences in temporal discrimination, following predictions from a recent theory of individual differences in WMC [22] and general theories of short-term memory [19]–[21], which propose that recall and recognition depend on discriminating memory representations by multiple attributes, foremost among which is temporal. Low-WMC individuals were less sensitive than high-WMC at discriminating the longer of two comparison intervals, across a range of duration differences and absolute durations. WMC-related differences in timing were also related to *gF*, but were not completely accounted for by it. These results are predicted most specifically by the dual-component WMC framework by Unsworth and Engle [22], but are also broadly consistent with the “executive attention view” of WMC (e.g., [1]; although not necessarily predicted by it). Results are also predicted by the temporal resolution power hypothesis by Troche and Rammsayer [46], which also argues for strong relationships among timing, WMC, and *gF*.
